# Impact of Depression and Anxiety on Outcomes Following Muscle-Preserving Transforaminal Lumbar Interbody and Fusion

**DOI:** 10.7759/cureus.105395

**Published:** 2026-03-17

**Authors:** Sahil Garg, Sean M Muir, Kyle Jamar, Alexa Dietrich, Patrick Young, Sonny Gill

**Affiliations:** 1 Orthopedic Research, Steadman Philippon Research Institute, Vail, USA; 2 Internal Medicine, OhioHealth, Columbus, USA; 3 Research, University of Colorado School of Medicine, Aurora, USA; 4 Orthopedics, Steadman Philippon Research Institute, Vail, USA; 5 Spine Surgery, The Steadman Clinic, Vail, USA

**Keywords:** functional disability, lumbar interbody fusion, mental health, patient-centered outcomes research, wiltse approach

## Abstract

Background: The impact of concurrent diagnoses of anxiety and depression has not been extensively studied in spine surgery. We hypothesize that a muscle-sparing technique may be associated with decreased postoperative pain and improved strength postoperatively compared to open techniques, decreasing the stress of the postoperative recovery process. Therefore, this study aims to assess the benefits that a modified muscle-preserving lumbar fusion technique (Wiltse transforaminal lumbar interbody and fusion (W-TLIF)) would have on this subset of patients.

Methods: One hundred twenty subjects who underwent a single-level W-TLIF were identified with a recorded diagnosis status associated with mental health. The subjects underwent surgery between January 2013 and January 2023 by the same surgeon at a single institution. Data was collected through a prospective registry and chart review. All subjects had a minimum of one-year follow-up. Mental health diagnosis, BMI, blood loss, operative duration, preoperative Short-Form 12 (SF-12) and Oswestry Disability Index (ODI) scores, and postoperative SF-12 and ODI scores were collected. Data was evaluated using SPSS version 29 (IBM Corp., Armonk, NY) and standard statistical modeling.

Results: One hundred twenty subjects with a confirmatory mental health diagnosis who underwent a single-level muscle-sparing TLIF were identified. Eighty-seven subjects had no diagnosis, six subjects were diagnosed with anxiety, 12 subjects were diagnosed with depression, and seven subjects were diagnosed with both anxiety and depression. Preoperative SF-12 was also included for an additional eight subjects for whom data was available. The mean total SF-12 score between cohorts was 78.5, 83.3, 74.6, and 85.9, respectively. The mean preoperative mental component scores were 48.2, 36.4, 42.0, and 42.2, respectively (p=0.002). The mean ODI scores between cohorts were 34.5, 45.2, 52.9, and 44.2, respectively (p=0.002). All subjects demonstrated statistically significant improvement in total SF-12 and ODI scores. There were no statistically significant differences in achievement between cohorts. The mean blood loss was 156.5 mL, 104.2 mL, 202.5 mL, and 142.9 mL, respectively, without statistical significance.

Conclusions: The subjects with a concurrent diagnosis of anxiety and/or depression can experience improvement postoperatively following W-TLIF. Despite having increased levels of preoperative disability, outcomes did not significantly differ from those without a mental health diagnosis. Blood loss, while not statistically significant, did trend lower in those with a diagnosis of anxiety and may be related to lower BMI. The preoperative optimization of anxiety and depression should likely still be considered in efforts to improve outcomes and patient satisfaction.

## Introduction

Depression and anxiety affect approximately one-third of patients undergoing spine surgery and are associated with inferior postoperative outcomes [1]. The relationship between chronic low back pain and psychological distress is bidirectional, with longitudinal data demonstrating that back pain predicts future depression while depression similarly predicts future back pain [2]. Large database analyses have shown that preoperative anxiety and depression negatively impact lumbar surgery outcomes, with an additive effect when both conditions are present [3]. Importantly, patients with depression demonstrate hypersensitivity to ischemic muscle pain, which may contribute to their inferior early postoperative outcomes [4].

Given this heightened pain sensitivity, surgical approaches that minimize tissue trauma and postoperative pain may be particularly beneficial for patients with anxiety and depression. The Wiltse paraspinal muscle-splitting approach exploits the natural intermuscular plane between the multifidus and longissimus muscles, reducing iatrogenic muscle injury and postoperative pain compared to traditional midline approaches [5,6]. A study comparing minimally invasive (MIS) to open transforaminal lumbar interbody and fusion (TLIF) demonstrated reduced narcotic requirements, shorter hospital stays, and faster return to work with muscle-sparing techniques [7]. These advantages may translate to improved outcomes in psychologically vulnerable patients who are predisposed to prolonged recovery and persistent pain.

Existing literature on mental health and MIS-TLIF outcomes suggests that while patients with psychological comorbidities have worse short-term results, they may still achieve meaningful long-term benefit. Yoo et al. found that patients with lower preoperative mental health scores had significantly less functional improvement at one year [8]. However, Goh et al. demonstrated in a five-year study that despite poorer early outcomes, patients with low mental component scores ultimately achieved comparable long-term results [9]. Whether the muscle-sparing benefits of the Wiltse approach specifically impact recovery in patients stratified by mental health status has not been evaluated. The primary aim of this study was to compare postoperative changes in Short-Form 12 (SF-12) component scores and Oswestry Disability Index (ODI) among patients stratified by mental health diagnosis (no diagnosis, anxiety alone, depression alone, or combined anxiety and depression) following single-level Wiltse transforaminal lumbar interbody and fusion (W-TLIF). We hypothesized that the muscle-sparing nature of W-TLIF would enable patients with anxiety and/or depression to achieve functional improvement, which was not significantly different from those without a mental health diagnosis.

## Materials and methods

Data was collected retrospectively through a prospectively collected registry and chart review. All subjects had a minimum of one-year follow-up. One hundred sixty subjects were included in the study. The subjects met the inclusion criteria if they had a reported mental health status confirmed within the electronic medical record system and reported BMI, intraoperative blood loss, operative duration, and patient-reported outcome measures (PROMs). PROMs encompassed a fractional mental component summary (MCS SF-12) and physical component summary (PCS SF-12) in addition to Short-Form 12 (SF-12) scores. PROMs were collected pre- and postoperatively. Any medical record that was incomplete was excluded from the study. Mental health diagnoses of anxiety and depression were confirmed through the electronic medical record based on the International Classification of Diseases, Tenth Revision (ICD-10) diagnostic codes assigned by treating physicians. Diagnosis was classified as present if documented at any point prior to the surgical date.

The subjects included in the study were those who underwent W-TLIF, between the ages of 18 and 65, and had failed nonoperative care (i.e., physical therapy, steroidal injections, and nonsteroidal anti-inflammatories) for their lumbar spine symptoms associated with radiculopathy and/or lumbar degeneration. Additionally, the exclusionary criteria included patients with tumors, patients with trauma, patients with infection, patients with a BMI of >40, patients with an HbA1c of >8, patients with anemia (hemoglobin {Hgb}: <9), patients with active or former smoking status, and patients under 18. The subjects underwent surgery between January 2013 and January 2023 by the same surgeon at a single institution. The W-TLIF was performed through a paraspinal muscle-splitting approach via the intermuscular plane between the multifidus and longissimus muscles. A unilateral facetectomy was performed to access the disc space, followed by discectomy, endplate preparation, interbody placement, and pedicle screw fixation. All patients followed a standardized perioperative protocol that included multimodal analgesia, early mobilization within 24 hours of surgery, and referral to outpatient physical therapy postoperatively. No mental health-specific perioperative interventions were utilized.

Patients with incomplete outcome data were excluded from analysis. Given the small subgroup sizes, multivariable adjustment was not performed. Baseline BMI, operative duration, and blood loss were compared across all cohorts to assess for possible confounders.

SPSS software version 29 (IBM Corp., Armonk, NY) was used to conduct the statistical analysis. The analysis of variance (ANOVA) was used to compare mean change scores between mental health diagnosis groups, with the F-statistic representing the ratio of between-group to within-group variability. P<0.05 was chosen as the threshold for statistical significance. Ethics permission for this study was given by the Vail Health Hospital Institutional Review Board (approval number: 2023-191).

## Results

A total of 120 subjects meeting the inclusion criteria were analyzed. The cohort consisted of 87 subjects with no mental health diagnosis, six with anxiety, 12 with depression, and seven with concurrent anxiety and depression (Table 1). Baseline analysis revealed significant disparities in preoperative mental health and functional status. Preoperative mental component summary (MCS) scores differed significantly across groups (p=0.002), with the anxiety cohort demonstrating the lowest baseline scores compared to the control group (36.4 versus 48.2) (Table 1). Notably, this psychological burden correlated with physical perception; preoperative Oswestry Disability Index (ODI) scores were significantly worse in patients with mental health diagnoses (p=0.002). Patients with depression alone reported the highest level of preoperative disability (mean ODI: 52.9), significantly higher than the control group (mean ODI: 34.5). Physical component summary (PCS) scores were comparable across all cohorts at baseline (p=0.674) (Table 1).

**Table 1:** Summary of PROs by Mental Health Status PCS, physical component summary; MCS, mental component summary; SF-12, Short-Form 12; ODI, Oswestry Disability Index; PROs, patient-reported outcomes

Characteristics	None (n=87)	Anxiety (n=6)	Depression (n=12)	Anxiety and depression (n=7)	F-value	P-value
Preoperative SF-12 PCS	34.4±9.1	31.7±6.1	32.0±6.3	32.1±6.6	5.087	0.674
Preoperative SF-12 MCS	48.2±10.5	36.4±8.9	38.9±11.3	42.2±11.1	0.919	0.002
Postoperative SF-12 PCS	37.2±10.1	42.6±6.7	34.5±9.2	38.2±9.4	1.388	0.434
Postoperative SF-12 MCS	48.9±11.2	55.6±8.3	43.8±14.4	44.7±14.0	2.236	0.170
Difference in SF-12 PCS	3.5±9.6	9.2±8.4	0.3±8.9	5.4±7.0	5.467	0.250
Difference in SF-12 MCS	4.2±12.7	5.7±8.2	3.7±11.5	-8.4±15.0	0.425	0.088
Preoperative ODI	34.5±14.9	45.2±3.3	52.9±16.1	44.3±13.3	2.617	0.002
Postoperative ODI	31.1±18.5	26.0±12.2	36.9±18.2	32.3±16.9	0.633	0.735
Difference in ODI	10.0±18.6	25.7±19.9	6.3±18.7	-4.9±36.0	-	0.055
Blood loss (mL)	156.5±153.0	104.2±55.7	202.5±173.4	142.9±114.3	-	0.595

There were no statistically significant differences in operative metrics between the cohorts, suggesting that the complexity of the surgical procedure and the intraoperative safety profile were not influenced by the patient's mental health status. Mean blood loss ranged from 104.2 mL (anxiety) to 202.5 mL (depression), but these differences did not reach statistical significance (p=0.595) (Table 1).

Postoperative ODI scores showed no significant difference between groups (p=0.735), indicating that patients with mental health comorbidities reached a similar final functional status to those without. While the anxiety cohort demonstrated robust improvement (mean change: 25.7), no cohort reached statistical significance. Similarly, while postoperative MCS scores improved across all groups, the difference in MCS improvement did not differ significantly between cohorts (p=0.088).

The analysis of variance (ANOVA) was used to compare mean change scores between mental health diagnosis groups, with the F-statistic representing the ratio of between-group to within-group variability. There was no significant difference in PCS change scores among mental health diagnosis groups following TLIF (F(3,108)=1.39; p=0.25), with a small effect size (η²=0.04). Change in MCS did not differ significantly by mental health diagnosis (F(3,108)=2.24; p=0.088), though a trend toward differential improvement was observed (η²=0.06).

## Discussion

There have been many studies evaluating surgical outcomes on mental and physical health, but few have evaluated individuals with depression and anxiety and their mental status's impact on outcomes. Our study examined the patients who were diagnosed with depression, anxiety, and anxiety and depression and their outcomes following a minimally invasive novel surgical technique, W-TLIF. This approach uses a smaller incision and dissection that occurs between the multifidus and longissimus planes, avoiding damage to the paraspinal muscles and downstream consequences. Our results demonstrated that patients with anxiety and depression, or a combination of both, had a lower preoperative SF-12 mental component summary score compared to those without a mental health diagnosis. Postoperative SF-12 mental component scores, although not statistically significant, exhibited improvement across all groups. However, patients with comorbid anxiety and depression had lower scores compared to patients without these conditions. The muscle-sparing W-TLIF appears to afford patients the opportunity for clinically meaningful improvement. However, the presence of anxiety and depression can potentially hinder the extent of improvement. While the data sets were limited in this study, the range of improvement for ODI in patients with a concurrent diagnosis was wide (36 points of variability in improvement), suggesting that physicians should traverse surgical expectation conversations preoperatively and cautiously. While surgical pathology may be addressed efficaciously, the secondary effects of surgery, even at one year postoperatively, may be perceived variably in this cohort of subjects.

Furthermore, it highlights that patients with a mental health diagnosis may be more susceptible, or have a lower threshold of mental tolerance, to the disabilities that back pain may cause. Our team has also demonstrated similar findings in cervical pathology, and we hypothesized that preoperative cognitive behavioral therapy may be beneficial for patients suffering from a mental health disorder.

Assessing functional status by Oswestry Disability Index (ODI) scores demonstrates that patients with comorbid anxiety and depression, or depression alone, have higher preoperative scores, indicating poorer functional status compared to those without a mental health diagnosis. Interestingly, patients with depression had the highest preoperative ODI score, which was greater in magnitude when compared to patients with anxiety or depression and anxiety. This preliminarily suggests that depression, when compared to anxiety, may modify a patient's perceived lumbar-specific disability to a greater extent [10]. While the mean values of ODI improvement in patients with depression and anxiety were lower than those without a mental health condition or anxiety, there was no observed statistical difference. While the subjects have the propensity to improve after surgery, it appears that, on average, those with depression and concurrent depression and anxiety did not achieve the cited minimal clinically important difference (MCID) improvements in ODI as frequently as mean improvements in ODI were 6.2 and -4.9 (>10 points) [11].

While variability in improvement does appear to exist at follow-up for patients with both depression and anxiety, this study is limited by sample size. The small sample sizes limit statistical power and increase susceptibility to a type II error. The nonsignificant difference between groups observed should be interpreted with caution, as the study may have been underpowered to detect clinically meaningful differences. The absence of statistically significant differences between groups should not be interpreted as evidence of equivalence until larger studies are completed. The study was unable to adjust for potential confounders such as psychiatric medical use, severity, or the duration of mental health diagnosis. As a single-surgeon, single-institution study, the results may not be generalizable to other surgical settings or patient populations. While limited, the existing literature does support that the subjects with depression and anxiety have the capacity to have significant improvement in disability at long-term follow-up [12,13]. Further investigation with larger sample sizes is advised to further examine the observed trends and associations.

## Conclusions

The subjects with a concurrent diagnosis of anxiety and/or depression may experience improvement postoperatively following W-TLIF. Patients with a concurrent diagnosis of anxiety and depression may experience greater variability in postoperative outcomes. Preoperative discussions in patients with formalized diagnoses of anxiety or depression should involve careful expectation setting, as there may be a propensity for these cohorts to have a greater degree of experienced disability. Blood loss, while not statistically significant, did trend lower in those with a diagnosis of anxiety and may be related to lower BMI. The preoperative optimization of anxiety and depression should likely still be considered in efforts to improve outcomes and patient satisfaction.

## References

[1] Strøm J, Bjerrum MB, Nielsen CV, Thisted CN, Nielsen TL, Laursen M, Jørgensen LB (2018). Anxiety and depression in spine surgery-a systematic integrative review. Spine J.

[2] Yang H, Hurwitz EL, Li J, de Luca K, Tavares P, Green B, Haldeman S (2023). Bidirectional comorbid associations between back pain and major depression in US adults. Int J Environ Res Public Health.

[3] Deshpande N, Hadi M, Mansour TR (2024). The impact of anxiety and depression on lumbar spine surgical outcomes: a Michigan Spine Surgery Improvement Collaborative study. J Neurosurg Spine.

[4] Bär KJ, Brehm S, Boettger MK, Boettger S, Wagner G, Sauer H (2005). Pain perception in major depression depends on pain modality. Pain.

[5] Wiltse LL, Bateman JG, Hutchinson RH, Nelson WE (1968). The paraspinal sacrospinalis-splitting approach to the lumbar spine. J Bone Joint Surg Am.

[6] Guiroy A, Sícoli A, Masanés NG, Ciancio AM, Gagliardi M, Falavigna A (2018). How to perform the Wiltse posterolateral spinal approach: technical note. Surg Neurol Int.

[7] Adogwa O, Parker SL, Bydon A, Cheng J, McGirt MJ (2011). Comparative effectiveness of minimally invasive versus open transforaminal lumbar interbody fusion: 2-year assessment of narcotic use, return to work, disability, and quality of life. J Spinal Disord Tech.

[8] Yoo JS, Hrynewycz NM, Brundage TS, Mogilevsky FA, Shah HC, Mehraban N, Singh K (2020). The influence of preoperative mental health on PROMIS physical function outcomes following minimally invasive transforaminal lumbar interbody fusion. Spine (Phila Pa 1976).

[9] Goh GS, Liow MH, Yeo W (2020). Patients with poor baseline mental health may experience significant improvements in pain and disability after minimally invasive transforaminal lumbar interbody fusion: a 5-year follow-up study. Clin Spine Surg.

[10] Hartman TJ, Nie JW, Zheng E, Oyetayo OO, MacGregor KR, Singh K (2023). Poor mental health scores correlate with inferior outcomes following minimally invasive transforaminal lumbar interbody fusion. Acta Neurochir (Wien).

[11] Copay AG, Glassman SD, Subach BR, Berven S, Schuler TC, Carreon LY (2008). Minimum clinically important difference in lumbar spine surgery patients: a choice of methods using the Oswestry Disability Index, Medical Outcomes Study questionnaire Short Form 36, and pain scales. Spine J.

[12] Goyal DK, Stull JD, Divi SN (2021). Combined depression and anxiety influence patient-reported outcomes after lumbar fusion. Int J Spine Surg.

[13] Goh GS, Liow MH, Yue WM, Tan SB, Chen JL (2021). Are patient-reported outcomes of minimally invasive transforaminal lumbar interbody fusion influenced by preoperative mental health?. Global Spine J.

